# Single-Mode-Tuned Tricolor Emissions of Upconversion/Afterglow Hybrids for Anticounterfeiting Applications

**DOI:** 10.3390/nano12183123

**Published:** 2022-09-09

**Authors:** Yanqing Hu, Songqi Li, Shijie Yu, Shuoran Chen, Yuyang Yan, Yan Liu, Yuanpeng Chen, Caosong Chen, Qiyue Shao, Yingshuai Liu

**Affiliations:** 1 School of Materials Science and Engineering, Suzhou University of Science and Technology, Suzhou 215009, China; 2 School of Materials Science and Engineering, Southeast University, Nanjing 211189, China; 3 School of Materials and Energy, Southwest University, Chongqing 400715, China

**Keywords:** upconversion, luminescence, afterglow, anticounterfeiting

## Abstract

This work presents a highly secure anticounterfeiting strategy based on upconversion/afterglow hybrids with tricolor emissions tuned by a single 975 nm laser. The hybrids are composed of NaYF_4_:Yb/Tm and NaYF_4_:Yb/Er microrods and CaS:Eu^2+^ afterglow phosphors. Under 975 nm excitation, the hybrids exhibit multicolor emissions from green to white by adjusting laser power and then emit red afterglow light when the 975 nm laser is off. Under synergistic excitation of the blue-green light emitted by Tm/Er microrods, the red afterglow emission not only has a strong initial intensity but also lasts for 3 s. Obvious trichromatic changes from green to white to red can be observed by the naked eye. A pattern printed by the hybrid ink exhibits tricolor emissions by laser adjustment and switch. This proves that upconversion/afterglow hybrids are an excellent candidate for anticounterfeiting applications with high-level security but a simple recognition method.

## 1. Introduction

Luxury goods, trademarks, medicines and currency are often faked by counterfeiters. Massive counterfeit activities cause an enormous economic loss to both customers and copyright owners [1]. Anticounterfeiting strategies can effectively prevent fraudulent goods from being traded in a market and thus suppress product counterfeiting acts [2]. As one of the security strategies, lanthanide-doped upconversion (UC) crystals absorb near-infrared photons and emit visible photons, showing significant advantages of low background fluorescence and strong resistance to photobleaching. For example, Yb/Er co-doped crystals with green emission have been applied in currencies and valuable documents [3]. Yet, the anticounterfeiting strategy with the single green UC emission is still likely to be imitated by using other substitutes with similar emissions.

In recent years, considerable progress has been made in developing UC materials with bicolor emissions [4,5,6]. Color-tunable emission is realized via different strategies including tunable lifetime multiplexing [7,8], adjusting laser power [9,10] and multimode fluorescence [11,12,13]. UC materials are outstanding in tuning multicolor emissions in multiple dimensions. These strategies greatly raise the security level of anticounterfeiting technology. Yet, triggering multicolor emission requires relatively complex equipment, such as time-gated decoding instrumentation, an ultrahigh power laser or multimode excitation sources. These complex identification methods limit commercial applications of anticounterfeiting materials. Hence, novel multicolor-emitting materials with a convenient recognition method deserve further investigation for anticounterfeiting applications. 

**Figure 1:**
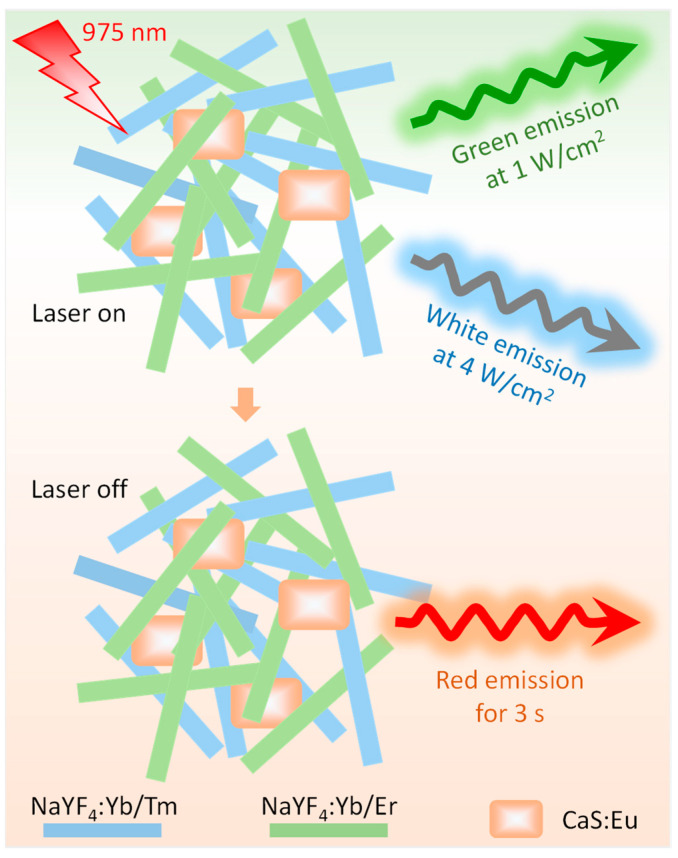
Schematic representation of tricolor emission of ternary hybrids consisting of NaYF_4_:Yb/Tm and NaYF_4_:Yb/Er microrods and CaS:Eu^2+^ phosphors induced by laser adjustment and switch.

In our previous studies, utilizing an opposite upconversion luminescence (UCL) thermal behavior, we have successfully realized various multicolor emissions through mixing both inert- and active-shell nanocrystals [14] and both small- and large-sized nanocrystals [15]. Moreover, based on an anomalous temperature-dependent UCL enhancement of small-sized nanocrystals, 975 nm excited multicolor emission has been achieved for single UC nanocrystals [16]. These novel UC materials can be used to produce color-tunable anticounterfeiting patterns and thus provide additional security features. A downside of some works is low initial UCL intensity. In addition, although UC and afterglow materials have made progress in the fields of optical encoding and biological imaging [17,18], they have not been applied in high-level anticounterfeiting technology. In this work, we report hybrids composed of UC microrods and CaS:Eu^2+^ phosphors. The strong blue-green emissions of the mixed microrods excite CaS:Eu^2+^ phosphors, emitting red afterglow light when the 975 nm laser is off (Figure 1). Trichromatic changes from green to white to red are triggered via 975 nm laser adjustment and switch. A color-tunable pattern printed with the hybrid ink exhibits high-level security for anticounterfeiting applications.

## 2. Results and Discussion

As a proof-of-concept experiment, we fabricated NaYF_4_:Yb/Tm and NaYF_4_:Yb/Er rodlike microcrystals. The microcrystals were synthesized via a solvothermal method [19] by introducing NaYF_4_ as host materials, Tm^3+^ and Er^3+^ ions as activators and Yb^3+^ ions as sensitizers. Scanning electron microscope (SEM) images showed a uniform morphology of the as-synthesized microcrystals (Figure S1a,b). These microcrystals were ~190 × 1600 nm in width and length on average. Powder X-ray diffraction (XRD) measurements confirmed a high crystallinity of the microrods with a single hexagonal (β) phase (Figure S2a). The detailed synthesis procedures of microrods are provided in the Supporting Information. In addition, Figure S1c shows that commercial CaS:Eu^2+^ afterglow phosphors are principally 8−18 μm in length with an irregular shape. XRD peaks of the CaS:Eu^2+^ phosphors can be well indexed into the standard card (PDF #08-0464), demonstrating a pure CaS phase (Figure S2b).

**Figure 2:**
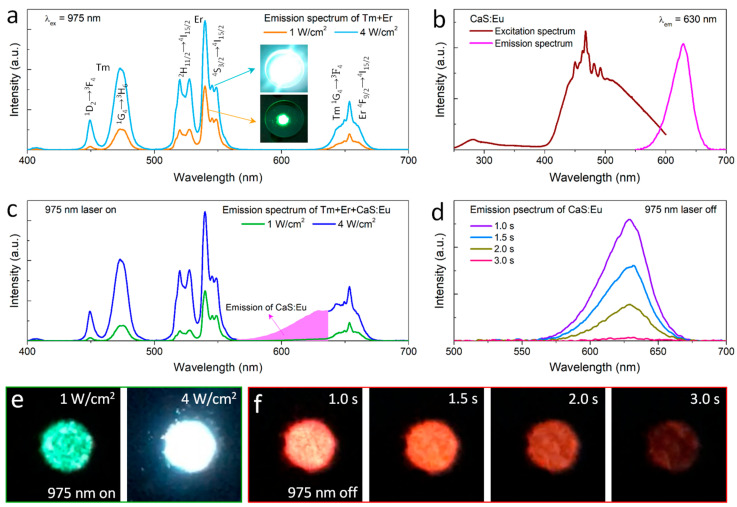
(**a**) UCL spectra of hybrids consisting of NaYF_4_:Yb/Tm and NaYF_4_:Yb/Er microrods. The insets show photographs of the solid hybrids under 975 nm excitation at various power densities. (**b**) Excitation spectrum of the commercially used CaS:Eu^2+^ phosphors with 630 nm emission. (**c**) Emission spectra of the hybrids composed of NaYF_4_:Yb/Tm, NaYF_4_:Yb/Er and CaS:Eu^2+^ under the 975 nm excitation at the different power densities. (**d**) Time-dependent emission intensities of hybrids at the 630 nm emission after the 975 nm laser is turned off. (**e**,**f**) Photos of solid hybrids when the 975 nm laser is on/off.

We measured the UCL spectra of hybrids consisting of both NaYF_4_:Yb/Tm and NaYF_4_:Yb/Er microcrystals (Figure 2a). The visible spectrum consists of blue, green and red bands, which mainly originate from Tm^3+ 1^G_4_ → ^3^H_6_, Er^3+ 2^H_11/2_, ^4^S_3/2_ → ^4^I_15/2_ and Tm^3+ 1^G_4_ → ^3^F_4_/Er^3+ 4^F_9/2_ → ^4^I_15/2_ transitions, respectively (Figures S3 and S4). Upon 975 nm excitation, the mixed microrods emit green light at a low-power density of 1 W/cm^2^ but exhibit blue emission at a high-power density of 4 W/cm^2^. In addition, the excitation spectrum was measured for CaS:Eu^2+^ afterglow phosphors with 630 nm emission. A broad excitation peak ranges from 400 nm to 600 nm, indicating that CaS:Eu^2+^ has strong absorption of blue-green light (Figure 2b). Hence, blue and green UCL can be used as the excitation wavelength of CaS:Eu^2+^ phosphors, emitting red afterglow light.

A perfect overlap between the emission spectrum of mixed microrods and the excitation spectrum of CaS:Eu^2+^ inspired us to design their hybrids, expected to exhibit color-shifting emissions. We next prepared ternary mixed crystals composed of NaYF_4_:Yb/Tm, NaYF_4_:Yb/Er and CaS:Eu^2+^ with a weight ratio of 3:2:4. The weight ratio of the constituent hybrids was optimized to balance emission intensities under 975 nm excitation at 4 W/cm^2^ for the generation of white emission color (Figure 2c). The laser-adjustment-induced color change is attributed to the relationship of I∝P^n^, where I, P and n are emission intensity, laser power and photon number, respectively [20]. Because the green emission of Er^3+^ is a two-photon process, while the blue emission of Tm^3+^ is a three-photon process (Figure S5), the blue emission increases faster with increasing laser power. After the 975 nm laser is turned off, the hybrids emit red light, which can last for 3 s (Figure 2d). The hybrid showed a significant color shift, from green to white to red via adjusting power and switching control. Photographs of the solid powder also demonstrate the color changes induced by the regulation and control of the 975 nm laser (Figure 2e,f). Accordingly, the calculated chromaticity coordinates (x, y) of the hybrid in the Commision Internationale de l’Eclairage (CIE) 1931 color space show an obvious laser regulation and control dependence, shifting from green (0.27, 0.52) to white (0.31, 0.33) with laser irradiation at various power density from 1 W/cm^2^ to 4 W/cm^2^ and then to red (0.66, 0.34) without laser irradiation (Figure S6). The color-shifting ability of the hybrid is quantifiably described by the chromaticity shift (∆C) induced by the laser on/off using the following equation [21].

(1) $$∆C=\sqrt{{\left(\mu_{O}^{’}-\mu_{I}^{’}\right)}^{2}+{\left(\nu_{O}^{’}-\nu_{I}^{’}\right)}^{2}+{\left(\omega_{O}^{’}-\omega_{I}^{’}\right)}^{2}}$$

where $\mu^{\prime }$ = 4*x/(*3 − 2*x* + 12*y)*, $\nu^{\prime }$ = 9*y/(*3 − 2*x* + 12*y)*, and $\omega^{\prime }$ = 1 − $\mu^{\prime }$ − $\nu^{\prime }$. $\mu^{\prime }$, $\nu^{\prime }$ and $\omega^{\prime }$ represent the chromaticity coordinates in the CIE 1976 uniform color space. *I* and *O* refer to the color coordinates under 975 nm laser regulation and control, respectively. The chromaticity shift (∆C) was calculated to be ≈ 0.10 for color change from green to white by adjusting the laser power and then to be ≈ 0.42 for color change from white to red when the 975 nm laser is off. The trichromatic changes respond quickly to the laser power regulation and on/off switching and also could be easily discerned by the naked eye.

**Figure 3:**
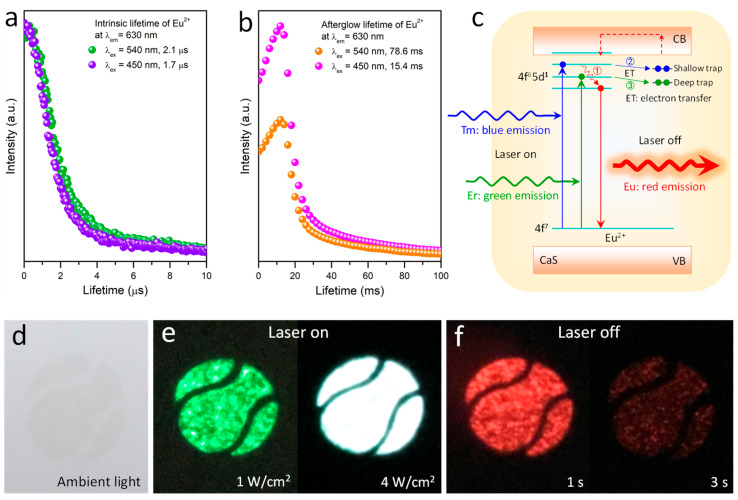
(**a**,**b**) Intrinsic lifetime and afterglow lifetime of CaS:Eu^2+^ under nanosecond and second pulsed laser excitations, respectively. (**c**) Schematic representation of excitation and luminescence dynamics of CaS:Eu^2+^ crystals. (**d**–**f**) Color changes of the printed pattern by using the hybrid ink under 975 nm irradiation at various power densities and when 975 nm laser is off.

To clarify the luminescence dynamics of Eu^2+^ ions, we measured lifetimes at the 630 nm emission under different excitation modes. Upon nanosecond pulsed laser excitations, the decay lifetime at 630 nm is 2.1 μs for 540 nm excitation and 1.7 μs for 450 nm excitation (Figure 3a). The result indicates that there is an initial fast decay corresponding to an intrinsic lifetime of Eu^2+^. The intrinsic lifetime is quite short due to a parity-allowed transition from 4f^6^5d^1^ to 4f^7^. The spontaneous radiation transitions of CaS:Eu^2+^ generate the red emission at 630 nm upon 450 nm and 540 nm excitations in the case of the 975 nm laser turned on (Figure 2c). Moreover, under second pulsed laser excitations, the decay lifetime of the 630 nm emission is 78.6 ms for 540 nm excitation and 15.4 ms for 450 nm excitation (Figure 3b). The decay lifetime reaches a millisecond level, demonstrating the red afterglow emission. This implies a photoinduced electron transfer from the Eu^2+^ excited state to the S^2^^−^ defect state, which is considered a trap. Furthermore, the lifetime of 540 nm excitation is longer than that of 450 nm excitation. The result suggests that 540 nm photon excited electrons are trapped to deep defect levels, while 450 nm photon excited ones are trapped to shallow defect levels (Figure 3c). In comparison to electrons in the deep defect level, the ones in the shallow defect level return to the Eu^2+^ excited state more quickly through a conduction band of CaS at room temperature. Hence, the red afterglow decay lifetime under the 450 nm excitation is shorter than that under the 540 nm excitation.

Single blue light excitation can efficiently generate red emission for CaS:Eu^2+^, emitting strong red light. However, the red afterglow time is short, and color changes from white to red cannot be observed by the naked eye. In contrast, the red emission excited by single green light is weak, but the red afterglow time can last for 3 s, which is enough for the naked eye to see the color change. Yet, under a synergistic excitation of both blue and green lights, not only the initial red emission intensity is strong, but also the persistence time is perfect. Therefore, the co-excitation of blue and green lights is necessary.

We further explored the application of the hybrids in the field of anticounterfeiting patterns. Taking advantage of the color tuning property dependent on laser regulating and control, the hybrids can be used to produce high-level security marking with additional authentication features. In addition, the color changes respond quickly to the laser adjustment and switch, and the recognition method is quite simple and convenient without complex reading instrumentations. For example, a printed pattern on the paper with the hybrid ink was nearly invisible in daylight (Figure 3d). The printed pattern appeared green and white on exposure to the 975 nm laser irradiation at various power but then exhibited red after the 975 nm laser was turned off (Figure 3e,f). An obvious emission color change from green to white to red was found through the 975 nm laser adjusting and switching. The result indicates that the color-tunable hybrids composed of mixed microrods and CaS:Eu^2+^ phosphors are an excellent candidate for anticounterfeiting applications with high-level security and a simple authentication method.

In comparison to our previous works [14,15,16,22], not only the initial luminescence intensity of hybrids is greatly improved due to the strong UCL of microrods, but also the color change is more abundant. The upconversion/afterglow hybrids further improve the security level and the practicability. In addition, the anticounterfeiting patterns printed with the upconversion/afterglow hybrids are recognized by a single 975 nm laser. Compared with other recognition methods, such as multimode fluorescence or time-gated decoding instrumentation, etc. [7,8,23,24,25], our single-mode identification method is a low-cost machine verification system. Undeniably, the preparation procedure of these materials is more complicated and difficult to forge by counterfeiters. Therefore, for anticounterfeiting materials that can be commercialized, we should comprehensively consider their security, production cost and machine verification cost.

## 3. Conclusions

In conclusion, novel anticounterfeiting materials were fabricated by directly mixing NaYF_4_:Yb/Tm and NaYF_4_:Yb/Er microrods and CaS:Eu^2+^ afterglow phosphors. Upon the 975 nm excitation at increased power density from 1 W/cm^2^ to 4 W/cm^2^, the hybrids exhibit multicolor emissions from green to white. Blue-green light in the white emission excites commercially used CaS:Eu^2+^ phosphors, emitting a red afterglow light after the 975 nm laser is turned off. The blue and green emissions originate from the UCL of Tm^3+^ and Er^3+^ ions, respectively. The red afterglow emission stems from the Eu^2+^ 4f^6^5d^1^ → 4f^7^ transitions of both relaxing electrons and trapped electrons. Under a synergistic excitation of both blue and green emissions, the red afterglow emission not only has a strong initial intensity but also lasts for 3 s. An obvious tricolor change from green to white to red can be observed by the naked eye, and the maximum chromaticity shift (∆C) reaches 0.42. The color-tunable hybrids are an excellent candidate for fabricating anticounterfeiting patterns with high-level security but a simple and convenient identification method.

## 4. Experimental Methods

### 4.1. Microrod Synthesis 

Hexagonal (β) phase NaYF_4_:Yb/Tm and NaYF_4_:Yb/Er microrods were synthesized using a solvothermal method. Detailed synthesis procedures of microcrystals are provided in the Supporting Information.

### 4.2. Characterization

SEM images were taken with a Sirion-400 field emission scanning electron microscope. XRD data were collected with a Shimadzu XD-3A X-ray diffractometer with Cu Kα radiation (λ = 1.5406 Å). UCL spectra were measured with a portable spectrometer (Maya2000Pro, Ocean Optics Co., LTD, Shanghai, China) using a continuous 975 nm diode laser as the excitation source. The diameter of the laser beam was ~8 mm at the powder sample position. Luminescent images of the printed patterns under 975 nm laser irradiation were taken by using an iPhone 7 digital camera (Apple Inc., Cupertino, CA, USA). Decay lifetime was tested with an FLS1000 fluorescence spectrometer (Edinburgh Instruments, Edinburgh, UK). The average lifetimes were determined via the following equation.

(2) $$\tau =\frac{1}{I_{0}}\int I\left(t\right)dt$$

where *I*(*t*) is the time-related emission intensity, and *I*_0_ is the maximum intensity.

## Supplementary Materials

The following supporting information can be downloaded at: https://www.mdpi.com/article/10.3390/nano12183123/s1. Detailed synthesis procedures of microcrystals, supporting figures including Figure S1: SEM images; Figure S2: XRD patterns; Figure S3: Simplified energy-level diagrams; Figure S4: UCL spectra; Figure S5: Double logarithmic plots; Figure S6: Color coordinates. Reference [25] is cited in the Supplementary Materials.
