# Supplementary material for: Single-Mode-Tuned Tricolor Emissions of Upconversion/Afterglow Hybrids for Anticounterfeiting Applications
Source: Nanomaterials (Basel). 2022 Sep 9;12(18):3123. doi: 10.3390/nano12183123 (PMC9503111; doi:10.3390/nano12183123)
Supplement: Supplementary file 1 [file nanomaterials-12-03123-s001.zip › nanomaterials-1855279-supplementary.pdf]

Supporting Information

Article

Single-Mode-Tuned Tricolor Emissions of Upconversion/Afterglow Hybrids for Anticounterfeiting Applications

Yanqing Hu [1,*], Songqi Li [1], Shijie Yu [2], Shuoran Chen [1], Yuyang Yan [1], Yan Liu [1], Yuanpeng Chen [1], Caosong Chen [1], Qiyue Shao [2,*] and Yingshuai Liu [3,*]

[1] School of Materials Science and Engineering, Suzhou University of Science and Technology, Suzhou 215009, China
[2] School of Materials Science and Engineering, Southeast University, Nanjing 211189, China
[3] School of Materials and Energy, Southwest University, Chongqing 400715, China
* Correspondence: author: yqh@usts.edu.cn (Y.H.); qiyueshao@seu.edu.cn (Q.S.); ysliu@swu.edu.cn (Y.L.)

Table of contents:

A. Methods

B. Figures

C. References

A. Methods

1. Chemical reagents

Yttrium(III) acetate hydrate (99.9%), ytterbium(III) acetate hydrate (99.9%), thulium(III) acetate hydrate (99.9%), erbium(III) acetate hydrate (99.9%), sodium hydroxide (NaOH, 98%), ammonium fluoride (NH_4F, 98%), sodium trifluoroacetate (Na-TFA, 98%), oleic acid (OA, 90%), 1-octadecene (ODE, 90%) were purchased from Sigma-Aldrich. $CaS:Eu^{2+}$ afterglow phosphors were provided by Dalian Luming Luminescent Technology Co., Ltd.

2. Synthesis of hexagonal microrods

Microrod crystals were synthesized by a solvothermal method [S1]. In a typical procedure for a synthesis of β-$NaYF_4$:Yb/Tm, or Er microrods, 1 mmol of lanthanide acetates (Y/Yb/Tm = 79.5:20:0.5, Y/Yb/Er = 78:20:2, mol%) were dissolved in 5 mL deionized (DI) water. In addition, NaOH (0.75 g, 18.75 mmol) was dissolved in 3.75 mL of DI water, followed by addition of 12.5 mL of OA and 12.5 mL of ethanol under vigorous stirring. Thereafter, an aqueous solution of NH_4F (2 mol/L, 2.5 mL) was added to form a turbid mixture. Subsequently, the dissolved rare earth acetate was added to above solution, kept stirring for 20 min. The resulting mixture was transferred into a 50-mL Teflon-lined autoclave and heated to 220 ^{o}C for 12 h. After cooling down to room temperature, the obtained microrods were isolated by centrifugation, washed with water and ethanol three times, and finally dried in an oven at 60 ^{o}C for further characterization.

3. Screen printing

30 mg of the hybrids mixing $NaYF_4$:Yb/Tm, $NaYF_4$:Yb/Er microrods and CaS:Eu phosphors with a weight ratio of 3:2:4 were dispersed into 100 μL terpineol. Subsequently, 24 mg of ethyl cellulose was added into the above upconversion/afterglow hybrids solution. The mixture was sonicated for 5 min in the hot water bath. Then, the as-prepared upconversion/afterglow fluorescent inks were printed on the paper through a 300 mesh counts screen to obtain various patterns.

B. Figures

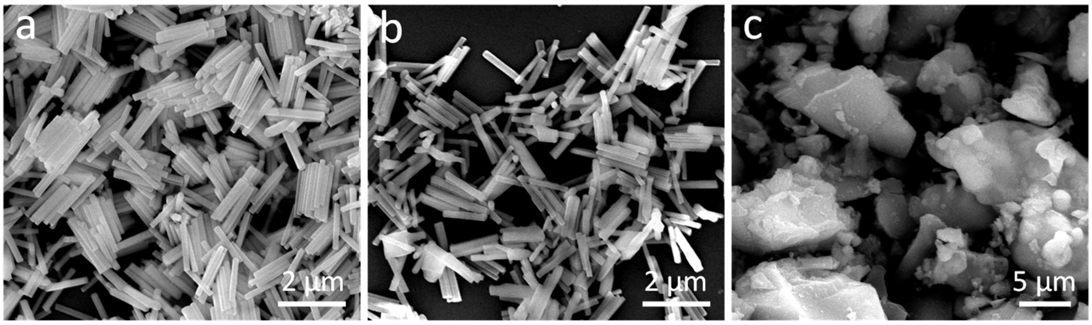

Figure S1. (a, b) SEM images of $NaYF_4$:20%Yb/0.5%Tm and $NaYF_4$:20%Yb/2%Er microrods (~190 × 1600 nm). (c) SEM image of commercial CaS:Eu^{2+} phosphors.

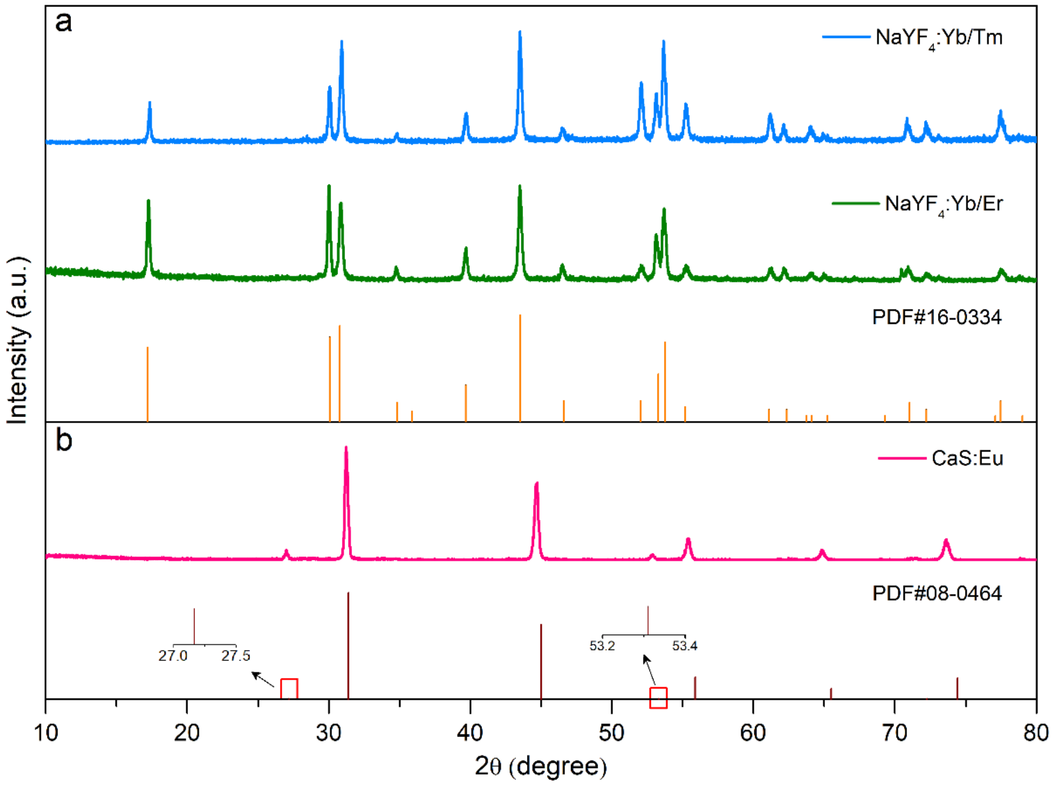

Figure S2. XRD patterns of $NaYF_4$:20%Yb/0.5%Tm, $NaYF_4$:20%Yb/2%Er microrods and commercial-used CaS:Eu^{2+} afterglow phosphors.

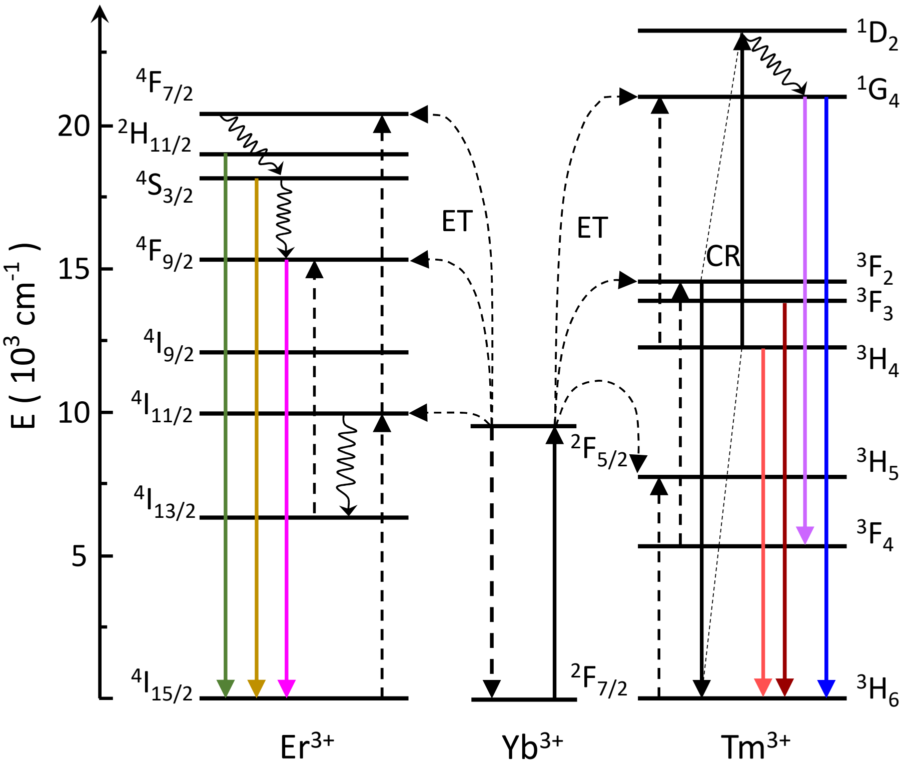

Figure S3. Simplified energy-level diagrams of Er−Yb and Tm−Yb system.

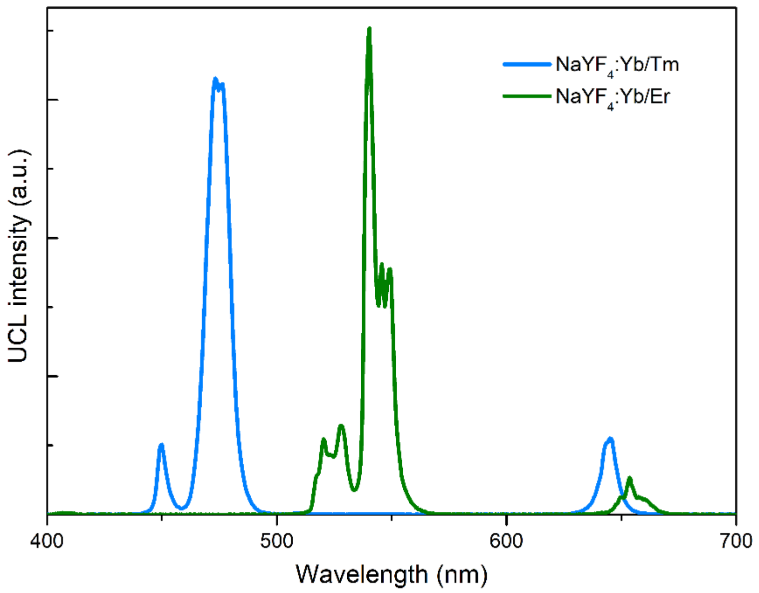

Figure S4. UCL spectra of $NaYF_4$:Yb/Tm and $NaYF_4$:Yb/Er microrods.

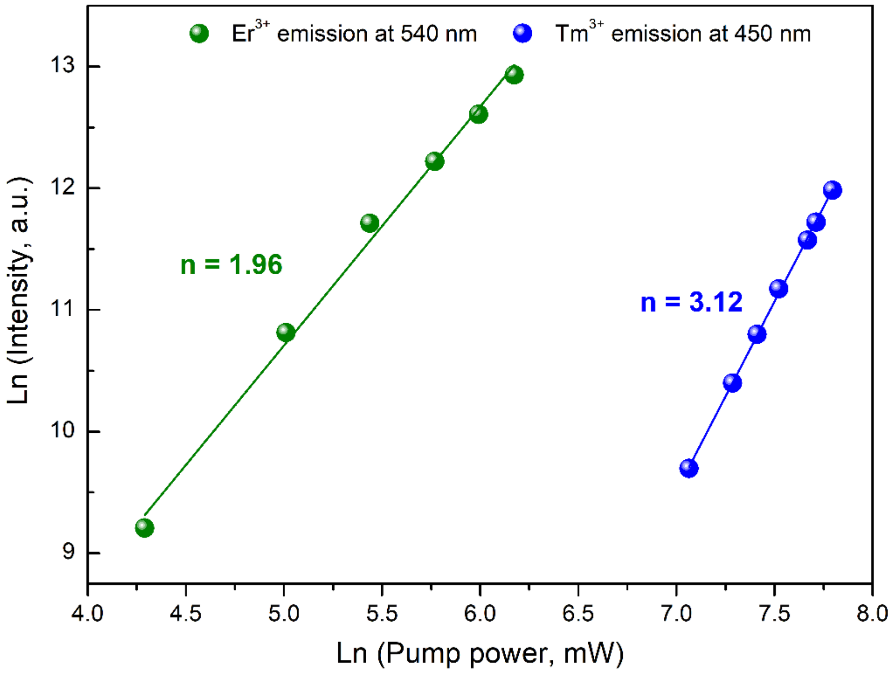

Figure S5. Double logarithmic plots of emission intensities vs laser power.

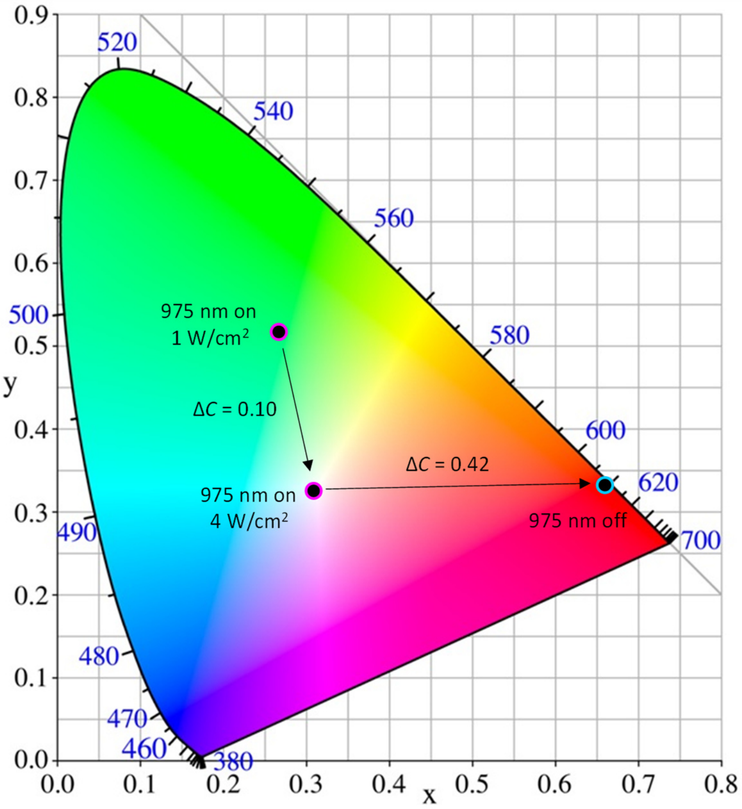

Figure S6. Color coordinates of hybrids consisting of $NaYF_4$:20%Yb/0.5%Tm, $NaYF_4$:20%Yb/2%Er microrods and CaS:Eu^{2+} afterglow phosphors in CIE chromaticity diagram upon 975 nm laser on-off.

C. References

[S1] Hu, Y.; Shao, Q.; Zhang, P.; Dong, Y.; Fang, F.; Jiang, J.: Mechanistic investigations on the dramatic thermally induced luminescence enhancement in upconversion nanocrystals. *J. Phys. Chem. C* **2018,** *122*, 26142−26152.
